# Evolution of Fc Receptor-Like Scavenger in Mammals

**DOI:** 10.3389/fimmu.2020.590280

**Published:** 2021-02-23

**Authors:** Maria Carolina Matos, Ana Pinheiro, José Melo-Ferreira, Randall S. Davis, Pedro José Esteves

**Affiliations:** ^1^ Centro de Investigação em Biodiversidade e Recursos Genéticos (CIBIO-UP), Centro de Investigação em Biodiversidade e Recursos Genéticos, InBIO, Laboratório Associado, Universidade do Porto, Vairão, Portugal; ^2^ Departamento de Biologia, Faculdade de Ciências, Universidade do Porto, Porto, Portugal; ^3^ Departments of Medicine, Microbiology, and Biochemistry & Molecular Genetics and the Comprehensive Cancer Center, University of Alabama at Birmingham, Birmingham, AL, United States; ^4^ CITS - Centro de Investigação em Tecnologias de Saúde, Cooperativa de Ensino Superior Politécnico e Universitário, CRL (CESPU), Gandra, Portugal

**Keywords:** FCR, FCRL, FCRLS, scavenger receptors, CD5L, evolution

## Abstract

Fc receptor-like (FCRL) molecules comprise a large family of receptors, homologous to the receptors for the Fc portion of immunoglobulins (FCR). Within this family, an unusual gene known to exist in mice, rats and dogs, termed *FCRLS*, encodes a chimeric protein with both Ig-like FCRL and type B scavenger-receptor cysteine-rich (SRCR)-like domains. In mice, *FCRLS* is located next to the *CD5L* and *KIRREL1* genes. Here, we show that the curious *FCRLS* gene is actually present across major mammalian groups, but its annotation is generally incorrect or absent. Anchored on mouse *FCRLS* and *FCRL2* genomic sequence alignments, phylogenetic analyses demonstrated that many mammalian sequences currently annotated as *FCRL2* cluster with *FCRLS*, supported by a conserved genetic synteny among organisms. This analysis shows that *FCRLS* is present in Rodentia, some Carnivora (Canidae and Ursidae), Chiroptera, Arctiodactyla, Proboscidae, and some Primata. Thus, the *FCRLS* most likely originated in a eutherian mammal ancestor since it is not present in Monotremata or Marsupialia. *FCRLS* has a peculiar distribution pattern across mammalian lineages, being present in some species, but absent in others from the same family, as in carnivores for example. The most parsimonious hypothesis to explain this *FCRLS* evolution is that it was convergently lost in several independent mammalian lineages. Analyses of branch-specific nucleotide evolutionary rates, show that *FCRL2* and *FCRLS* have similar ranges of rates across mammals, suggesting that both genes have crucial, but separate functions in the immune system. Bayesian estimates of evolutionary rates for *FCRLS* in mammalian lineages revealed that carnivores display the highest mutation rate after rodents. Additionally, positive diversifying selection was detected for both *FCRL2* and *FCRLS*. Our results show that the presence of the *FCRLS* gene is older and more widespread across mammals than previously thought and appears to be functional, being under positive selection. Its precise physiologic role should thus be investigated.

## Introduction

Members of the classical Fc receptor (FCR) family that bind IgG and IgE belong to the greater immunoglobulin superfamily and are responsible for preserving the complex balance between cellular and humoral immunity in higher vertebrates (1, 2). Mammals possess different classes of FCRs, which correlate with their genetic, molecular, and structural diversity as well as ligand affinity. Accordingly, the number of isotype-specific FCRs has increased throughout vertebrate evolution most likely in parallel with the expansion of Ig-isotypes (1, 3). The FCRL molecule family is related to an ancient multigene family that is suspected to be linked by a common ancestor to the classical FCRs. The relationship between the FCRs and FCRLs is evident in their genetic organization, similar composition and structure of encoded extracellular domains, and the use of cytoplasmic tyrosine-based signaling elements (4, 5). In addition to their structural similarities, in primates, such as macaques, orangutans, chimpanzees and humans, all of the classical *FCRs* and *FCRL* are located on chromosome 1, implicating their evolution from a common ancestor (1).

The *FCRL* family comprises genes encoding type I transmembrane proteins with various Ig-like extracellular domains, multiple isoforms and allelic variations, preferential B lineage expression, and autonomous or dual signaling properties, with most displaying immunoreceptor tyrosine-based activating (ITAM) and/or inhibitory (ITIM) motifs in their cytoplasmic tails (1, 2, 4). For example, FCRL2 encodes a transmembrane protein with four extracellular Ig-like domains and a cytoplasmic tail with ITIM and ITAM-like sequences that is expressed by memory B cells (6). Despite having a conserved sequence motif in one Ig domain subtype that suggests Ig-binding capability, these receptors do not appear to possess the same binding properties as the classical FCR (4). Instead, many of the ligands for these molecules have remained elusive (7). However, recent discoveries have demonstrated the ability of FCRL3 to bind secretory IgA (8), FCRL4 to bind IgA, FCRL5 to bind IgG, and FCRL6 to interact with MHC class II molecules (9–11). Eight different *FCRL* genes have been identified in mammalian genomes including *FCRL1–6*, *FCRLA* and *FCRLB* (5, 12). However, not all eight *FCRL* genes are simultaneously present in every species. From a functional standpoint, FCRL molecules appear to primarily dampen immune responses (1). This hypothesis is supported by an overrepresentation of ITIMs in their cytoplasmic tails and numerous studies that have shown an association between autoimmune disorders and genetic variation in *FCRL* genes, more specifically the involvement of *FCRL3* polymorphisms in autoimmunity (1, 7, 13–16).

In contrast to the abundance of *FCRL* genes present in the human, canine, and elephant genomes, only five orthologues were identified in mice: *FCRL1* and *FCRL5* on chromosome 3 and *FCRL6*, *FCRLA* and *FCRLB* on chromosome 1 (1). Together with these genes, an atypical gene, known as *FCRLS*, was identified, which does not exist in humans, but is also present in the rat and dog genomes (1, 3). This gene encodes a mosaic protein that lacks a transmembrane-encoding segment and includes an exon encoding a type B scavenger receptor cysteine rich (type B-SRCR) domain. These features make FCRLS unique as an Ig-like/SRCR-B chimeric protein (3, 17). Scavenger receptors possess various biological functions, forming a structurally diverse and large family of proteins. Some of these SRCR proteins localize on the cell surface as transmembrane molecules and may bind multiple ligands that aid in the removal of non-self or altered-self molecules through adhesion, endocytosis, phagocytosis, and signal transduction (18, 19). Scavenger receptors are mainly expressed by myeloid cells, and Brown and Goldstein (20) were the first to report their activity in macrophages, when investigating the formation of lipid-laden macrophages in atherosclerotic plaques (18, 21). The *FCRLS* gene in mice is also highly expressed by microglial cells in non-hematopoietic organs. In fact, a recent gene expression and proteomic analysis identified *FCRLS* as a differentially expressed gene (DEG), that can discriminate central nervous system (CNS)-derived microglial cells from peripheral monocytes/macrophages (22–24). Validation of FCRLS as a translated protein that is involved in CNS-related pathology (glioma) further indicates its value as a distinguishing marker of monocyte populations and supports the premise that this gene has functional relevance in the immune system (22).

**Figure 1:**
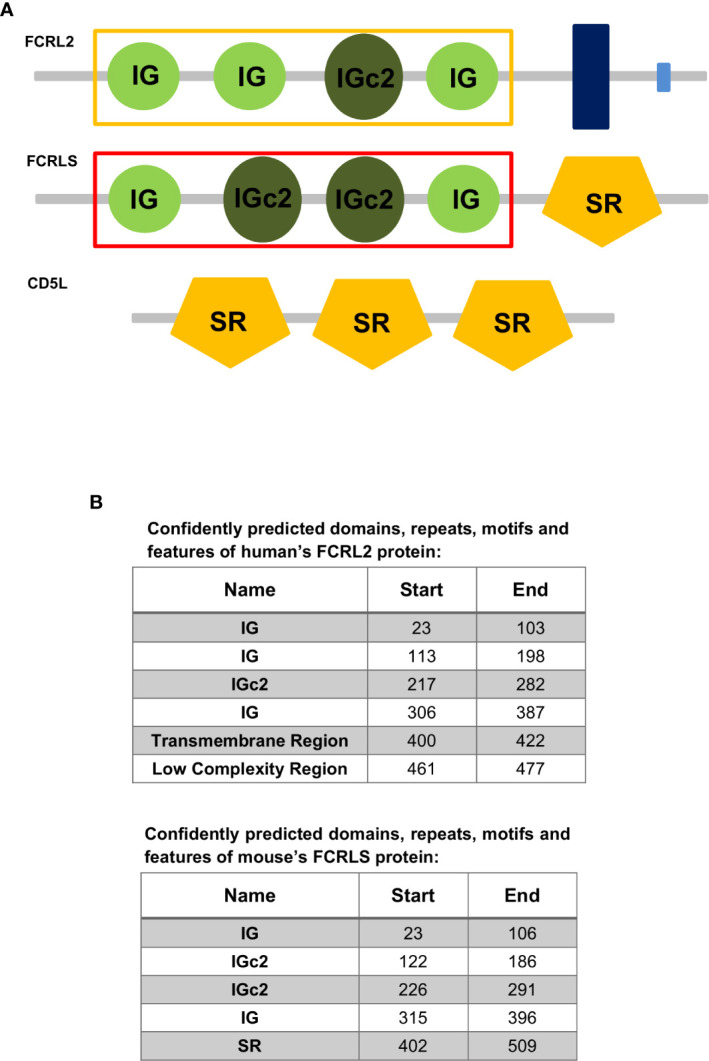
**(A)** Representation of the domain organization of the Fc receptor-like protein 2 (FCRL2) of *Homo sapiens* and the Fc receptor-like S, scavenger receptor (FCRLS) of *Mus musculus* and the CD5 molecule like (CD5L) of *Homo sapiens*. The yellow and red rectangles surrounding the IG and IGc2 domains signalize the domains used to construct the ML tree. IG, Immunoglobulin domain; IGc2, Immunoglobulin C-2 Type domain; SR, Scavenger receptor Cys-rich (SRCR) domain; Dark Blue Rectangle, Transmembrane region; Light Blue Rectangle, Low complexity region. **(B)** Confidently predicted domains, repeats, motifs and features of Fc receptor-like protein 2 (FCRL2) of *Homo sapiens* and the Fc receptor-like S, scavenger receptor (FCRLS) of *Mus musculus* (http://smart.embl-heidelberg.de/).

Proteins harboring scavenger receptor cysteine-rich (SRCR) motifs, more recently classified as class I scavenger receptors, constitute a group of scavenger receptors that engage in the development of the immune system, and regulate innate and adaptive immune responses (18, 25). Members of the SRCR typically possess one or multiple repeats of a cysteine-rich extracellular domain and can be membrane-bound or secreted (25). A member of this class is the CD5L, a soluble glycoprotein, also known as apoptosis inhibitor expressed by macrophages (AIM) due to its first identified function (26, 27). This protein is involved in a wide range of biological functions, and circulates in serum at relatively high concentrations. The main cellular source of CD5L is tissue macrophages and it has been implicated in various models of inflammatory diseases, such as systemic lupus erythematosus (26–28). CD5L possesses three scavenger domains very similar to the one present in FCRLS (the SRCR-like domain of FCRLS shows 62% amino acid identity with mouse AIM/CD5L) (17). Given that *FCRLS* is generally found immediately adjacent to the *CD5L* gene, has the opposite orientation of *FCRL1* and *FCRL5*, and appears to have a chimeric genetic structure that encodes a soluble protein, it is likely that this gene has resulted from an ancient recombination event involving a member of the *FCRL* family and *CD5L* (4). A common feature preserved among the *FCR* and *FCRLs* is a split signal peptide encoded by two exons, with the second exon consistently constituting 21 bp, which gives rise to the second half of the leader sequence (2, 29–31). This characteristic 21 bp second exon is also present in *FCRLS*. While the *CD5L* gene also possesses a split signal peptide, in contrast to the *FCR*/*FCRL* genes and *FCRLS*, the second half of the signal peptide is encoded by a 35 bp exon (26). The similarities in the protein domains of FCRLS, FCRLs and CD5L support the hypothesis that recombination events may be responsible for the genetic makeup and origin of *FCRLS* (**Figure 1**).

The *FCRL* family presents high genetic and structural diversity and a peculiar evolutionary history that requires further clarification. Here, we applied bioinformatics tools to gain a more comprehensive view of the distribution of these genes in a group of selected mammalian genomes. This analysis demonstrates that the *FCRLS* gene is actually present in most of major mammalian families and is either incorrectly annotated as *FCRL2-like* in most organisms or simply not annotated. These findings provide important new insights into the evolution of *FCRLS*.

## Methods

### Sequences

Publicly available gene sequences for mammalian genomes annotated as *FCRL2*, *FCRL2-like* or *FCRLS* were obtained through BLASTn (Standard Nucleotide BLAST) searches, using the *Mus musculus*, *Rattus norvegicus*, and *Canis lupus familiaris FCRLS* gene sequence and the *Homo sapiens* FCRL2 gene sequence as query. Searches were conducted in the NCBI’s GenBank (http://www.ncbi.nlm.nih.gov/genbank/) and Ensembl (https://www.ensembl.org/index.html) genome databases (32). Sequences that contained premature stop codons and/or frameshifting insertions or deletions were discarded from the analysis. The obtained sequences were aligned using ClustalW (33) in BioEdit (34) software and the resulting alignment was modified manually as needed as to respect reading frame correctness and exon boundaries. The resulting alignments are available as **Supplementary Material** (**Supplementary Data 1**). Subsequently, the identified gene sequences synteny was analyzed to ensure that all sequences were in fact orthologous genes. In total, we analyzed 81 sequences of 74 species, including representatives of Rodentia, Primata, Carnivora, Chiroptera, Artiodactyla, Lagomorpha, and Proboscidea (the respective accession numbers are given in **Supplementary Table 1**).

### Phylogenetic Analysis

The phylogenetic relationships of the *FCRL2* and *FCRLS* genes were analyzed in a Maximum Likelihood (ML) framework, using the GTR+G+I model of nucleotide substitution, in MEGA version X 10.1.6 software (35). Node support was estimated using 1000 bootstrap replicates of ML trees. *FCRL3 s*equences of five mammals’ were used to root the tree.

### Genomic Synteny Analysis

To determine relative syntenic positions among genomes, gene loci were identified from the NCBI (https://www.ncbi.nlm.nih.gov/gene/) and Ensembl (https://www.ensembl.org/index.html) databases and the sizes of the genes, including the distance between the neighbouring genes, were calculated and used to construct a map in scale of the loci (support data given in **Supplementary Table 2**) using the approach described in (1, 36). For each species, we used the transcriptional variant with the same exons, when present, or the only sequence available.

### Nucleotide Evolutionary Rates

The identified *FCRLS* and *FCRL2* genes were separated into two distinct alignments, which were used to infer evolutionary rates with the Bayesian method implemented in BEAST v1.10.4 (37), under a strict molecular clock and under an uncorrelated lognormal relaxed clock (38). This relaxed clock model estimates the evolutionary rate for each branch of the tree, thus accounting for the possibility of extreme rate heterogeneity, while the strict model clock assumes constancy of evolutionary rates across branches. The analyses were calibrated setting normally distributed priors for the time of the most recent common ancestor, retrieved from the TimeTree database (39), of seven monophyletic clades for *FCRLS* (Boreotheria, 96 million years ago (Mya); Scrotifera, 79 Mya; Primatomorpha, 76 Mya; Rodentia, 73 Mya; Yangochiroptera, 53 Mya; Caniformia, 46 Mya; and Bovidae, 24.6 Mya) and eight monophyletic clades for *FCRL2* (Boreotheria, 96 million Mya; Euarchonta, 82 Mya; Ferae, 75 Mya; Primata, 73.8 Mya; Caniformia, 46 Mya; Simiiformes, 43.2 Mya; Feliformia, 40 Mya; and Lemuroidea, 38 Mya), and a standard deviation of 2. Posterior probabilities were determined using Yule tree priors and GTR+G nucleotide substitution models. Independent runs of 10,000,000 generations were performed, and convergence was assessed using Tracer v1.7 (40). Final estimates were based on the combined results of three replicate runs, discarding the first 10% as burn-in. Posterior trees were summarized using TreeAnnotator v1.10.4, included in the BEAST v1.10.4 package. Marginal likelihood estimation (MLE), using the path sampling approach, available in BEAST, was performed to calculate Bayes Factors and determine which clock model (strict clock or uncorrelated lognormal relaxed clock) best fitted the data.

### Codon-Based Analyses of Positive Diversifying Selection

To determine whether both genes, *FCRLS* and *FCRL2*, evolved under similar evolutionary regimes, we compared the rate per-site of nonsynonymous substitution (dN) to the rate per-site of synonymous substitutions (dS) for each gene separately in a maximum likelihood (ML) framework using three different methods. As each of the methods employs unique algorithms, and hence has advantages and drawbacks, we only considered those codons identified by a minimum of two of the ML methods as being positively selected codons (PSC) (41–43). Using MEGA version X 10.1.6 software (35), a Neighbor-joining (NJ) tree was obtained for each gene dataset to be used as the working topology, with the p-distance substitution model and the complete deletion option to handle gaps and missing data. Generally, the topology of the used trees reflected the accepted topology for mammals.

Two alternative models were compared in CODEML (PAMLX) (44, 45), one allowing codons to evolve under positive selection (dN/dS>1), M8, and one that does not (dN/dS ≤ 1), M7. The analyses were performed with the F3x4 model of codon frequencies and were run twice to guarantee congruous results. The models were compared using a likelihood ratio test (LRT) with 2 degrees of freedom (46, 47). For the M8 model, codons under positive selection were determined using a Bayes Empirical Bayes approach (BEB) (48) and considering a posterior probability of >90%.

We then used two methods for detecting positive selection accessible on the DataMonkey web server (49): the Random Effect Likelihood model (REL) and the Mixed Effects Model of Evolution (MEME). Both methods of analysis were run three times to ensure consistent results. The best fitting nucleotide substitution model was first determined by the automatic model selection tool available on the server.

To verify that recombination was not providing a false assumption of positive selection (50–53), we used the GARD method from the DataMonkey web server (49) to screen the datasets. The results did not show evidence of recombination.

## Results

By screening 74 mammalian genomes, we identified multiple species where two annotated copies of the *FCRL2* gene, that differed both in genome location and orientation, were apparently present. By aligning these two gene copies, differences among the genomic sequences suggested that the two *FCRL2* gene copies were, in fact, two distinct genes. To test this hypothesis, we performed gene alignments, constructed phylogenetic trees, and performed evolutionary rate variation analyses.

### Phylogenetic and Evolutionary Analysis

In this study, 81 genomic sequences for *FCRLS*-related genes, identified through BLASTn, were phylogenetically analyzed using a Maximum-Likelihood method (ML) and the GTR+G+I model of nucleotide substitution.

**Figure 2:**
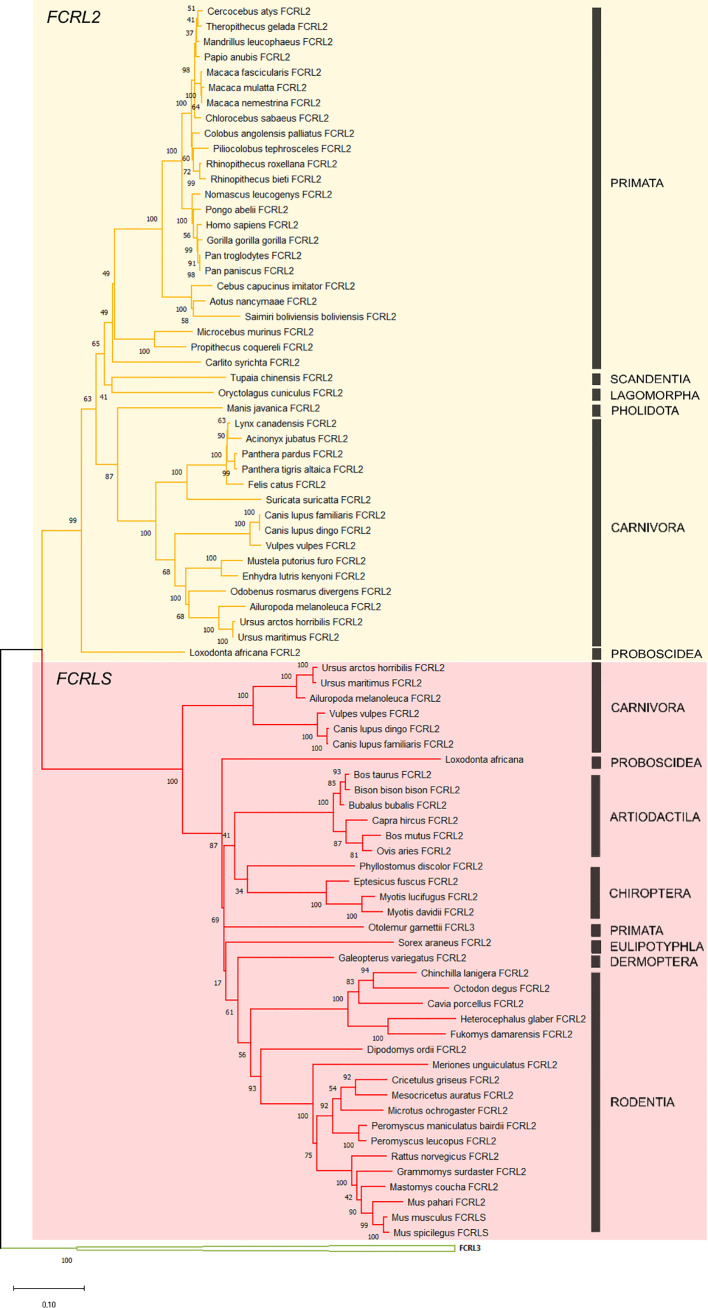
Phylogenetic tree of the *FCRL2* and *FCRLS* genes. Maximum likelihood (ML) method and the GTR+G+I model of nucleotide substitution were used to assemble the phylogenetic tree. Some groups were collapsed for simplification. The tree is color-coded: in yellow are the *FCRL2* branches (n=43), in red the *FCRLS* branches (n=38) and in green the *FCRL3* branches that were used to root the tree. Bootstrap values are indicated to the left of each node. Black bars indicate mammalian orders.

The obtained ML phylogenetic tree clearly supports two distinct groups, one clustering *FCRL2* genes (n=43) and a second encompassing the rodent *FCRLS* genes (n=38) supported by bootstrap values of 99 and 100, respectively (**Figure 2**). Considering that the scavenger portion of the FCRLS and transmembrane region of FCRL2 sequences could not be properly aligned, we constructed the ML phylogenetic tree using only the Ig-like domains of *FCRL2* and *FCRLS*.

The genes found to cluster with the rodent *FCRLS* (hence referred to as *FCRLS*) were mostly annotated as *Fc Receptor-like 2* or *Fc Receptor-like protein 2*, but in *Otolemur garnettii, FCRLS* was actually annotated as *Fc receptor-like protein 3*.** A deeper analysis of each gene sequence and its predicted encoded protein domains, revealed a resemblance between these genes and *FCRL2*, including shared similar Ig-like domains (**Figure 1**), which may explain their misannotation. The major difference in this cluster resides in the type B-SRCR scavenger portion of the genes, which is conserved in *FCRLS* sequences throughout species (76% and 68% of nucleotide and amino acid similarity, respectively)

With this analysis, we have identified the presence of *FCRLS* in Proboscidea, Arctiodactyla, Carnivora, Chiroptera, Primata, Dermoptera, and Eulipotyphla, confirming its presence in ancestral species such as *Loxodonta africana.* No significant blasts were obtained for the *FCRLS* gene in the Monotremata and Marsupialia genomes suggesting that this gene is not present in these groups. Interestingly, the *FCRLS* gene seems to be absent in several independent lineages. In Primata this gene is present in *Otolemur garnettii*, a Lorisiform, but is absent among most other primates. In Carnivora, this gene is present in Ursidae and Canidae families, but is absent in Felidae, Mustelidae, Odobenidae, and Herpestidae. We were also unable to find *FCRLS* for *Oryctolagus cuniculus*, the European rabbit, which is a Lagomorph, the sister group to Rodentia. Additionally, we searched for *FCRLS* remains in the scaffolds containing *FCRLS* flanking genes, *KIRREL1* and *CD5L*, for the species for which we obtained no significant blasts. Overall, the coverage for these scaffolds is very good ranging 7x to 411x. In addition to this unusual evolutionary pattern, the carnivore *FCRLS* genes appear to cluster in a well-supported group (bootstrap value=93, **Figure 2**) at a basal position relative to other mammals.

### Genomic Synteny Analysis

**Figure 3:**
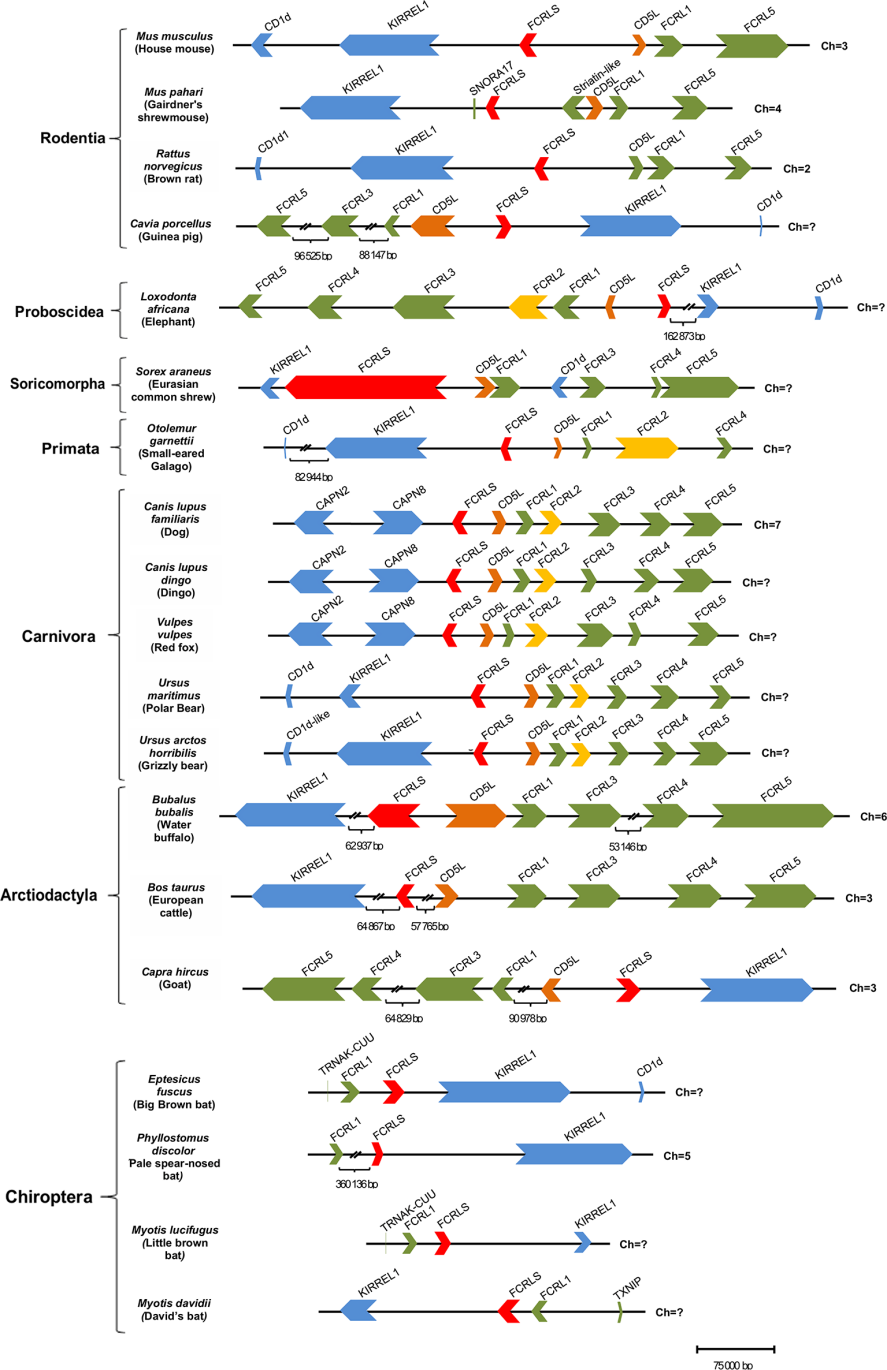
Structure of the *FCRL* gene family locus among a panel of mammalian genomes. Each horizontal line corresponds to the chromosome on which the *FCRL* and flanking genes are located. Genes are color-coded: *FCRL* genes are shown in green except *FCRL2* that is in yellow, *FCRLS* is in red, *CD5L* is in orange, flanking genes on the opposite side of the *FCRL* cluster are in blue, and flanking genes next to the *FCRL* cluster are also depicted in green.

Of the 74 mammalian genomes analyzed here, 38 harbour the *FCRLS* gene (**Figure 2**). For most, the *FCRLS* gene is located between *KIRREL1* and *CD5L* and is in the opposite orientation of the *FCRL* gene locus, revealing a shared synteny (**Figure 3**). Given that *FCRLS* generally shares the same orientation as *KIRREL1*, it is possible that these genes may have undergone an ancient translocation event. However, there are exceptions. For example, in Canidae, this gene has the same orientation as most mammals; yet, instead of being flanked by *KIRREL1*, *FCRLS* is located between *CAPN8* and *CD5L*, both of which have the same orientation as the *FCRL* genes. The other exception observed is in Chiroptera, where the *CD5L* gene is lacking and there is generally a very limited repertoire of *FCRL* genes. When present, the *FCRL* genes typically have the same orientation as *FCRLS* and *KIRREL1*.

Further analysis of the genomic synteny, revealed that the *Mus musculus FCRLS* gene is composed of 8 exons, but the *Rattus norvegicus FCRLS* gene is composed of 7 exons. It also became clear that the *FCRLS* gene has the same approximate genetic length in most species with two exceptions. The first example is *Sorex araneus* (scaffold coverage of 120X) in which *FCRLS*, despite having the same number of exons as *M. musculus*, has large introns that yield the most expansive *FCRLS* genetic length among mammals. The second is *Bubalus bubalis* (scaffold coverage 62X) in which *FCRLS* is comprised of 10 exons, two more than in *M. musculus*, and has a large first intron.

### Evolutionary Rates

**Figure 4:**
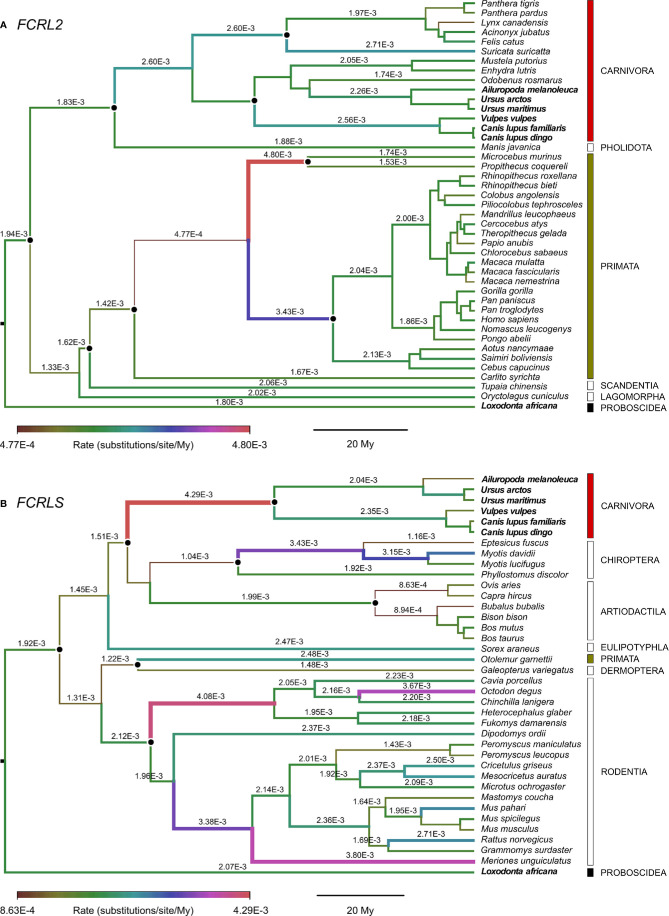
Branch-specific evolutionary rates (units of substitutions/site/My) for **(A)** *FCRL2* (n=43) and **(B)** *FCRLS* (n=38) inferred using an uncorrelated lognormal relaxed clock implemented in Beast v1.10.4 and plotted with FigTree. The median of the high posterior density distribution is shown above branches; some rates were omitted for convenience of display. The thickness and colour of branches vary according to the inferred rate, and black dots indicate the calibrated nodes. Red, green and black bars indicate mammalian orders represented in both *FCRL2* and *FCRLS* analyses, while empty bars depict orders with data from one of the genes. Bold species names indicate those present in both *FCRL2* and *FCRLS* analyses.

The Bayes Factors (BF) showed decisive support against strict clock models in both *FCRL2* and *FCRLS* (log_10_(BF) = 40 and 76 respectively), suggesting evolutionary rate variation across lineages. The mean nucleotide evolutionary rate estimated across all tree branches was slightly higher for *FCRLS* than for *FCRL2* (2.22 x 10^-3^ and 1.90 x 10^-3^ substitutions/site/My, respectively) but with an overlap of the 95% high posterior distribution (HPD) intervals (2.02 x 10^-3^ – 2.42 x 10^-3^, and 1.73 x 10^-3^ – 2.06 x 10^-3^ substitutions/site/My for *FCRLS* and *FCRL2* respectively). In *FCRLS*, higher rates were estimated for some Rodentia (e.g. 4.08 x 10^-3^ substitutions/site/My), Carnivora (4.29 x 10^-3^ substitutions/site/My) and Chiroptera (e.g. 3.43 x 10^-3^ substitutions/site/My) (**Figure 4**). Contrastingly, Artiodactyla showed the smaller evolutionary rates. In *FCRL2*, branches with high rates were estimated in Primata (e.g. 4.80 x 10^-3^ substitutions/site/My or 3.43 x 10^-3^ substitutions/site/My) (**Figure 4**).

### Analyses of Positive Diversifying Selection

Through the comparison of the CODEML contrasting models, we found evidence of positive selection acting for both *FCRL2* and *FCRLS*, with the model allowing sites to evolve under positive selection (M8) showing a significantly better fit than the model that did not (M7) for both genes (**Table 1**).

**Table 1:** Phylogenetic tests of positive selection in the mammalian sequences used for this study.

	Test of Selection				Sites under selection identified by different methods^a^			
Dataset	lnL M7 (neutral)/ lnL M8 (selection)	-2lnΔLc	Significance	p_s_;ω_s_^b^	PAML M8	REL	MEME	PSC^c^
**FCRL2**	-15613,9/ -15508,4 (1,0068)	211	**(p<0,001)	0,03; 3,02	23, 42, 59, 63, 70, 83, 85, 86, 87, 88, 89, 90, 91, 92, 93	23, 42, 55, 56, 59, 63, 68, 70, 73, 85, 86, 87, 88, 89, 90, 91, 92, 93, 101, 131, 243, 348, 401, 413, 433, 434, 438, 446	2, 5, 10, 11, 14, 15, 16, 17, 19, 23, 37, 39, 42, 45, 47, 55, 56, 59, 63, 64, 68, 70, 73, 76, 82, 84, 85, 86, 87, 89, 91, 92, 93, 94, 101, 115, 131, 176, 183, 184, 202, 213, 243, 246, 276, 278, 285, 294, 310, 318, 349, 350, 354, 377, 385, 397, 401, 410, 413, 426, 433, 438, 446, 454, 461, 464, 473	23, 42, 55, 56, 59, 63, 68, 70, 73, 85, 86, 87, 88, 89, 90, 91, 92, 93, 101, 131, 243, 401, 413, 433, 438, 446
**FCRLS**	-20072,4/ -20021,3 (1,0025)	102,2	**(p<0,001)	0,01; 2,27	62, 88, 94, 101, 162, 504, 505, 506, 508, 509	27, 41, 44, 74, 94, 162, 397	3, 20, 35, 41, 44, 45, 62, 64, 74, 75, 89, 94, 97, 100, 104, 108, 125, 143, 157, 158, 159, 160, 162,185, 190, 210, 240, 263, 274, 276, 304, 320, 324, 334, 337, 351, 361, 373, 397, 411, 423, 465, 490, 505, 506, 507, 508, 509, 510	41, 44, 62, 74, 94, 162, 397, 505, 506, 508, 509

^a^Codons identified by more than one ML method are underlined.

^b^p_s_ = proportion of the sites under selection; ω_s_ = estimated dN/dS of the sites under selection in M8.

^c^PSC (Positively Selected Codons) – only the codons identified by at least two of the ML methods were considered to be positively selected codons.

Comparing the sites identified by each of the three used methods, 26 PSCs were identified for *FCRL2* and 12 PSCs were identified for *FCRLS* (**Table 1**). Positive selection seems to act with greater incidence in the first Ig domain of *FCRL2*, 19 of the 26 identified PSCs locate to this domain (**Table 1**). As for *FCRLS*, positive selection acts essentially in the first and last protein domains with 5 PSCs locating to the first Ig domain and 5 PSCs locating to the scavenger domain.

To understand the biological significance of the detected PSC, functional studies are required in order to establish the residues responsible for the interaction with ligands and to determine the three-dimensional structure of both proteins.

## Discussion

From the phylogenetic results obtained, it is clear that what is commonly annotated as a second *FCRL2* copy is actually *FCRLS*, a gene previously described only in the rodent and canine genomes (**Figures 2** and **3**). Our analyses of evolutionary variation rates along with the phylogenies of *FCRL2* and *FCRLS* show a similar pattern of rate variation, strongly suggesting that like *FCRL2*, *FCRLS* is also a functional gene. The highest evolutionary rates for *FCRLS* were observed in rodent branches; however, our analyses also suggest that *FCRLS* is a fast-evolving gene in Carnivores. On the contrary, the lowest rates were found in Artiodactyla (**Figure 4**). These results suggest that distinct selective evolutionary pressures on *FCRLS* are at play and have governed the evolution of this gene among different groups of mammals, as is supported by the positive diversifying selection analysis (**Table 1**). Through these analyses, we uncovered strong positive selection to be acting for both genes, which further supports our hypothesis that both have crucial roles in immunity. Both genes display PSCs in the first Ig domain, which leads us to believe that this domain is particularly important in the function or conformation of the protein, but more studies will have to be conducted. For *FCRLS*, we also found PSCs in the scavenger domain that, once more, support our hypothesis that this domain is crucial for the protein function.

**Figure 5:**
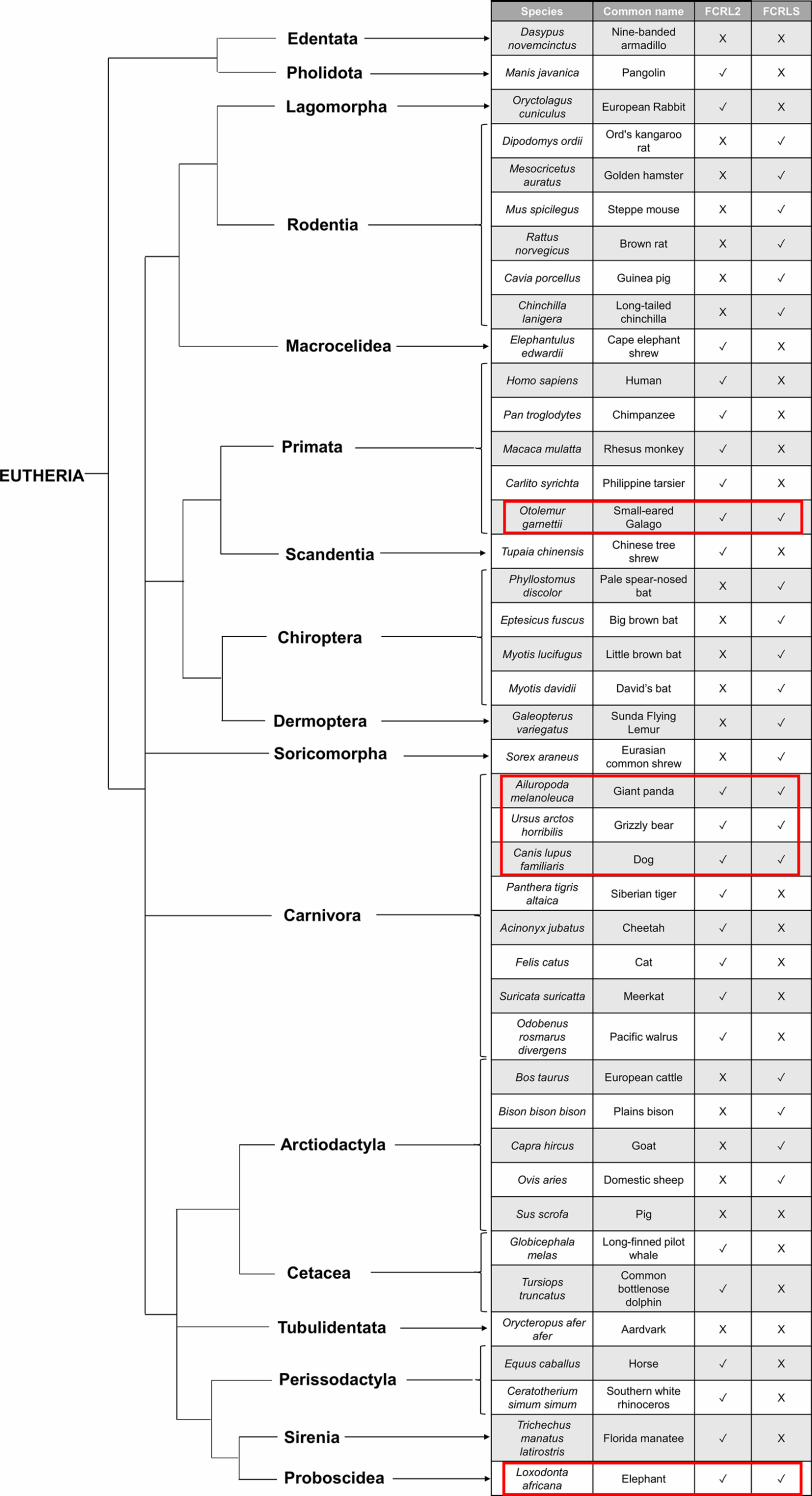
Species tree illustrating the evolutionary pattern of *FCRLS* and *FCRL2*. The red rectangles surround the species that retain the *FCRL2* and the *FCRLS* gene.

The synteny of the *FCRLS* locus is conserved across mammalian lineages, though some lineages show changes in this locus. This is especially the case in Chiroptera, which have lost most *FCRL* genes but retain *FCRLS* in the opposite orientation when compared to most mammals. Furthermore, most species retained either the *FCRL2* gene or the *FCRLS* gene, with very few exceptions where both genes are present (**Figure 5**). It is unlikely that the loss of one of the genes could have eventually resulted from a functional redundancy of the two genes as *FCRL2* and *FCRLS* display very distinct expression patterns. *FCRL2* is known to be expressed as a type I transmembrane protein on human memory B cells (6), having significant expression the lymph node, spleen and appendix of human tissues (https://www.ncbi.nlm.nih.gov/gene/79368/?report=expression). *FCRLS* appears to encode a soluble protein that is expressed by microglial cells in mice (4, 22, 24) and has significant transcript expression in adult mouse tissues including mammary gland, subcutaneous fat pad, cerebral cortex, frontal lobe and bladder (https://www.ncbi.nlm.nih.gov/gene/80891/?report=expression). The expression in mouse brain tissues has been confirmed at the protein level (22, 24), leading us to believe that FCRLS plays an important role in the immune responses of the brain. Thus, these disparate protein structures and cellular distributions imply distinctly different functions for FCRL2 and FCRLS. In organisms lacking the *FCRLS* gene, its function could possibly be ensured by other scavenger receptors rather than the *FCRL2* gene.

The evolutionary scenario that seems to accommodate all of our observations is that *FCRLS* originated by recombination between a *FCRL* gene and a gene with a scavenger domain, in an eutherian mammal ancestor. The *FCRLS* gene is not present in Monotremata or Marsupialia, but is present in an ancient African mammal, the elephant (*Loxodonta africana*), indicating that the *FCRLS* gene appeared before the eutherian mammal diversification. The most accepted hypothesis for the emergence of *FCRLS* is that *CD5L* was involved in the recombination that originated the *FCRLS* gene (4), but we were unable to prove that, since our BLAST analysis of the scavenger portion of the *FCRLS* did not produce a significant match with a *CD5L* gene (divergence higher than 30%). This could be due to the accumulation of structural and conformational changes of this domain in order to make the FCRLS protein more efficient. Alternatively, the recombination donor gene with the scavenger domain was lost during the recombination process.

Interestingly, and despite having identified the *FCRLS* gene for representatives of most mammalian orders, this gene is not present in several independent lineages. In Primata this gene is present in *Otolemur garnettii*, a Lorisiform, but is absent among most other primates. This suggests that the gene was present in the Primata ancestral but was lost in the Lemuriformes (Lorisiformes sister group) at circa 60 million years ago and also in the Haplorrhini at circa 74 million years ago (39, 54). For Carnivora, this gene is present in Ursidae and Canidae families but is absent in Felidae, Mustelidae, Odobenidae, and Herpestidae, suggesting *FCRLS* was lost independently in these lineages. We were also unable to find *FCRLS* for *Oryctolagus cuniculus*, the European rabbit, which is a Lagomorph, the sister group to Rodentia that were separated at 65 million years ago (39). The most parsimonious hypothesis to accommodate this unusual evolutionary pattern is that the *FCRLS* emerged in an eutherian mammal ancestor and was then convergently lost in several independent mammalian lineages. An alternative scenario would involve the independent appearance of the *FCRLS* at least 3 times during mammalian evolution, which seems highly unlikely given the conserved gene structure and synteny observed among mammals.

At this point, it is unclear whether the loss reflected unnecessary redundant function or was instead accompanied by functional replacement from other genes. Regardless, the identification of *FCRLS* in many mammalian families’ hints at an important immunological function for this receptor, especially given its identification as a microglial DEG marker, which suggests an essential role in neurological immune responses.

## Conclusion

The results obtained in this work show that the *FCRLS* gene was wrongly annotated as *FCRL2* in the majority of the screened mammalian genomes. The *FCRLS* is most likely the result of a recombination between an *FCRL* gene and a scavenger domain gene. *FCRLS* is older than previously thought and it was already present before the Eutherian mammal’s radiation. It is present at least in Rodentia, some Carnivora (Canidae and Ursidae), Chiroptera, Arctiodactyla, Proboscidae and in a primitive primate, *Otolemur garnettii*. The *FCRLS* gene distribution pattern across all mammals is very unusual with the loss of this gene at different time points in three main mammalian groups: Primata, Carnivora and Lagomorpha. The variation in evolutionary rates and the identified PSCs across mammalian lineages and species suggests that distinct selective pressures governed the evolution of this gene, compatible with a functional role in immunity, which requires clarification. The FCRLS molecule has a SRCR-type B domain that may be indicative of its scavenger functions and properties. The broad distribution of this gene with indication of positive selection pressure opens new research avenues that should be explored by expression and functional studies.

## Data Availability Statement

The original contributions presented in the study are included in the article/**Supplementary Material**. Further inquiries can be directed to the corresponding author.

## Author Contributions

MM analyzed the data and wrote the manuscript. AP and JM-F analyzed and discussed the data. RD discussed the data. PE conceived the study. All authors contributed to the article and approved the submitted version.

## Funding

This work was funded by national funds through FCT—Foundation for Science and Technology—under the project PTDC/BIA-OUT/29667/2017. FCT also supported the post-doctoral fellowships of AP (ref. SFRH/BPD/117451/2016), the FCT Investigator grant of PE (IF/00376/2015) and the Scientific Stimulus contract of JM-F (CEECIND/00372/2018). RD was funded in part by NIH/NIAID award AI110553.

## Conflict of Interest

The authors declare that the research was conducted in the absence of any commercial or financial relationships that could be construed as a potential conflict of interest.
