# Supplementary material for: Evolution of Fc Receptor-Like Scavenger in Mammals
Source: Front Immunol. 2021 Feb 23;11:590280. doi: 10.3389/fimmu.2020.590280 (PMC7940838; doi:10.3389/fimmu.2020.590280)
Supplement: Supplementary file 2 [file DataSheet_2.docx]

Supplementary Material

# Supplementary Data

**Supplementary Data 1.** FcRL2 and FCRLS sequence alignments.

# Supplementary Tables

**Supplementary Table 1.** Genbank accession number of the sequences used in this study

| **Species** | **Common name** | **Order-Family** | **Accession number** | **Data base annotation** | **Proposed annotation** |
| --- | --- | --- | --- | --- | --- |
| *Loxodonta africana* | Elephant | Proboscidea – Elephantidae | ENSLAFG00000026489 | Novel gene | Fc receptor-like S, scavenger receptor (FCRLS) |
| *Galeopterus variegatus* | Sunda flying lemur | Dermoptera – Cynocephalidae | XM_008591706.1 | Fc receptor-like protein 2 | Fc receptor-like S, scavenger receptor (FCRLS) |
| *Otolemur garnettii* | Small-eared Galago | Primates – Galagidae | XM_012808500.1 | Fc receptor-like protein 3 | Fc receptor-like S, scavenger receptor (FCRLS) |
| *Dipodomys ordii* | Ord's kangaroo rat | Rodentia – Heteromyidae | XM_013030542.1 | Fc receptor-like protein 2 | Fc receptor-like S, scavenger receptor (FCRLS) |
| *Peromyscus maniculatus bairdii* | Prairie deer mouse | Rodentia – Cricetidae | XM_006976429.1 | Fc receptor-like protein 2 | Fc receptor-like S, scavenger receptor (FCRLS) |
| *Peromyscus leucopus* | White-footed mouse | Rodentia – Cricetidae | XM_028860473.1 | Fc receptor-like protein 2 | Fc receptor-like S, scavenger receptor (FCRLS) |
| *Cricetulus griseus* | Chinese hamster | Rodentia – Cricetidae | ENSCGRT00000004507.1 | Fc receptor-like protein 2 | Fc receptor-like S, scavenger receptor (FCRLS) |
| *Microtus ochrogaster* | Prairie vole | Rodentia – Cricetidae | XM_013349808.2 | Fc receptor-like protein 2 | Fc receptor-like S, scavenger receptor (FCRLS) |
| *Mesocricetus auratus* | Golden hamster | Rodentia – Cricetidae | XM_013120555.2 | Fc receptor-like protein 2 | Fc receptor-like S, scavenger receptor (FCRLS) |
| *Grammomys surdaster* | Common Grammomys | Rodentia – Muridae | XM_028753313.1 | Fc receptor-like protein 2 | Fc receptor-like S, scavenger receptor (FCRLS) |
| *Mus musculus* | House mouse | Rodentia – Muridae | NM_030707.3 | Fc receptor-like S, scavenger receptor (FCRLS) | ~~---------------~~ |
| *Mus pahari* | Gairdner's shrewmouse | Rodentia – Muridae | XM_021196430.2 | Fc receptor-like protein 2 | Fc receptor-like S, scavenger receptor (FCRLS) |
| *Mus spicilegus* | Steppe mouse | Rodentia – Muridae | ENSMSIG00000021565 | Fc receptor-like S, scavenger receptor | ~~---------------~~ |
| *Mastomys coucha* | Southern multimammate mouse | Rodentia – Muridae | XM_031374264.1 | Fc receptor-like protein 2 | Fc receptor-like S, scavenger receptor (FCRLS) |
| *Rattus norvegicus* | Brown rat | Rodentia – Muridae | NM_001107702.1 | Fc receptor-like 2 (FCRL2) | Fc receptor-like S, scavenger receptor (FCRLS) |
| *Meriones unguiculatus* | Mongolian gerbil | Rodentia – Muridae | XM_021655017.1 | Fc receptor-like protein 2 | Fc receptor-like S, scavenger receptor (FCRLS) |
| *Cavia porcellus* | Guinea pig | Rodentia – Caviidae | XM_003466640.3 | Fc receptor-like protein 2 | Fc receptor-like S, scavenger receptor (FCRLS) |
| *Chinchilla lanigera* | Long-tailed chinchilla | Rodentia – Chinchillidae | XM_013522273.1 | Fc receptor-like protein 2 | Fc receptor-like S, scavenger receptor (FCRLS) |
| *Heterocephalus glaber* | Naked mole-rat | Rodentia – Bathyergidae | XM_013077364.2 | Fc receptor-like protein 2 | Fc receptor-like S, scavenger receptor (FCRLS) |
| *Octodon degus* | Common degu | Rodentia – Octodontidae | XM_023721634.1 | Fc receptor-like protein 2 | Fc receptor-like S, scavenger receptor (FCRLS) |
| *Fukomys damarensis* | Damaraland mole-rat | Rodentia – Bathyergidae | XM_019210120.2 | Fc receptor-like protein 2 | Fc receptor-like S, scavenger receptor (FCRLS) |
| *Sorex araneus* | Eurasian common shrew | Soricomorpha – Soricidae | XM_012935337.1 | Fc receptor-like protein 2 | Fc receptor-like S, scavenger receptor (FCRLS) |
| *Bubalus bubalis* | Water buffalo | Artiodactyla – Bovidae | XM_006079646.2 | Fc receptor-like protein 2 | Fc receptor-like S, scavenger receptor (FCRLS) |
| *Bos taurus* | European cattle | Artiodactyla – Bovidae | XM_024989820.1 | Fc receptor-like protein 2 | Fc receptor-like S, scavenger receptor (FCRLS) |
| *Bison bison bison* | Plains bison | Artiodactyla – Bovidae | XM_010843762.1 | Fc receptor-like protein 2 | Fc receptor-like S, scavenger receptor (FCRLS) |
| *Bos mutus* | Wild yak | Artiodactyla – Bovidae | XM_005910592.1 | Fc receptor-like protein 2 | Fc receptor-like S, scavenger receptor (FCRLS) |
| *Capra hircus* | Goat | Artiodactyla – Bovidae | XM_018046529.1 | Fc receptor-like protein 2 | Fc receptor-like S, scavenger receptor (FCRLS) |
| *Ovis aries* | Domestic sheep | Artiodactyla - Bovidae | XM_027978013.1 | Fc receptor-like protein 2 | Fc receptor-like S, scavenger receptor (FCRLS) |
| *Phyllostomus discolor* | Pale spear-nosed bat | Chiroptera – Phyllostomidae | XM_028530691.1 | Fc receptor-like protein 2 | Fc receptor-like S, scavenger receptor (FCRLS) |
| *Eptesicus fuscus* | Big brown bat | Chiroptera – Vespertilionidae | XM_008153191.2 | Fc receptor-like protein 2 | Fc receptor-like S, scavenger receptor (FCRLS) |
| *Myotis lucifugus* | Little brown bat | Chiroptera – Vespertilionidae | XM_014462771.2 | Fc receptor-like protein 2 | Fc receptor-like S, scavenger receptor (FCRLS) |
| *Myotis davidii* | David’s bat | Chiroptera – Vespertilionidae | XM_015564232.1 | Fc receptor-like protein 2 | Fc receptor-like S, scavenger receptor (FCRLS) |
| *Ailuropoda melanoleuca* | Giant panda | Carnivora – Ursidae | XM_019810202.2 | Fc receptor-like protein 2 | Fc receptor-like S, scavenger receptor (FCRLS) |
| *Ursus arctos horribilis* | Grizzly bear | Carnivora – Ursidae | XM_026487325.1 | Fc receptor-like protein 2 | Fc receptor-like S, scavenger receptor (FCRLS) |
| *Ursus maritimus* | Polar Bear | Carnivora – Ursidae | XM_008700756.1 | Fc receptor-like protein 2 | Fc receptor-like S, scavenger receptor (FCRLS) |
| *Canis lupus dingo* | Dingo | Carnivora – Canidae | XM_025430734.1 | Fc receptor-like protein 2 | Fc receptor-like S, scavenger receptor (FCRLS) |
| *Canis lupus familiaris* | Dog | Carnivora – Canidae | XM_005622658.3 | Fc receptor-like protein 2 | Fc receptor-like S, scavenger receptor (FCRLS) |
| *Vulpes vulpes* | Red fox | Carnivora – Canidae | XM_026003285.1 | Fc receptor-like protein 2 | Fc receptor-like S, scavenger receptor (FCRLS) |
| FCRL2 |  |  |  |  |  |
| *Canis lupus familiaris* | Dog | Carnivora – Canidae | XM_849766.5 | Fc receptor-like protein 2 | ~~---------------~~ |
| *Canis lupus dingo* | Dingo | Carnivora – Canidae | XM_025430743.1 | Fc receptor-like protein 2 | ~~---------------~~ |
| *Vulpes vulpes* | Red fox | Carnivora – Canidae | XM_026003286.1 | Fc receptor like 2 (FCRL2) | ~~---------------~~ |
| *Ailuropoda melanoleuca* | Giant panda | Carnivora – Canidae | XM_011237545.3 | Fc receptor-like protein 2 | ~~---------------~~ |
| *Ursus arctos horribilis* | Grizzly bear | Carnivora – Canidae | XM_026487376.1 | Fc receptor-like protein 2 | ~~---------------~~ |
| *Ursus maritimus* | Polar Bear | Carnivora – Canidae | XM_008700763.1 | Fc receptor-like protein 2 | ~~---------------~~ |
| *Lynx canadensis* | Canada lynx | Carnivora – Felidae | XM_030301836.1 | Fc receptor like 2 (FCRL2) | ~~---------------~~ |
| *Panthera pardus* | Leopard | Carnivora – Felidae | XM_019438558.1 | Fc receptor like 2 (FCRL2) | ~~---------------~~ |
| *Panthera tigris altaica* | Siberian tiger | Carnivora – Felidae | XM_015538445.1 | Fc receptor like 2 (FCRL2) | ~~---------------~~ |
| *Felis catus* | Cat | Carnivora – Felidae | XM_019822375.2 | Fc receptor-like protein 2 | ~~---------------~~ |
| *Acinonyx jubatus* | Cheetah | Carnivora – Felidae | XM_027048363.1 | Fc receptor-like protein 2 | ~~---------------~~ |
| *Callorhinus ursinus* | Northern fur seal | Carnivora – Otariidae | XM_025860574.1 | Fc receptor-like protein 2 | ~~---------------~~ |
| *Eumetopias jubatus* | Steller sea lion | Carnivora – Otariidae | XM_028090103.1 | Fc receptor-like protein 2 | ~~---------------~~ |
| *Zalophus californianus* | California sea lion | Carnivora – Otariidae | XM_027611961.1 | Fc receptor-like protein 2 | ~~---------------~~ |
| *Odobenus rosmarus divergens* | Pacific walrus | Carnivora – Odobenidae | XM_004415960.1 | Fc receptor-like 2 (FCRL2) | ~~---------------~~ |
| *Suricata suricatta* | Meerkat | Carnivora – Herpestidae | XM_029935888.1 | Fc receptor like 2 (FCRL2) | ~~---------------~~ |
| *Mustela putorius furo* | Domestic ferret | Carnivora – Mustelidae | XM_004775774.2 | Fc receptor-like 2 (FCRL2) | ~~---------------~~ |
| *Enhydra lutris kenyoni* | Sea otter | Carnivora – Mustelidae | XM_022490697.1 | Fc receptor-like protein 2 | ~~---------------~~ |
| *Homo sapiens* | Human | Primates – Hominidae | NM_030764.4 | Fc receptor like 2 (FCRL2) | ~~---------------~~ |
| *Pan troglodytes* | Chimpanzee | Primates – Hominidae | XM_524903.6 | Fc receptor like 2 (FCRL2) | ~~---------------~~ |
| *Pan paniscus* | Bonobo | Primates – Hominidae | XM_003821020.3 | Fc receptor like 2 (FCRL2) | ~~---------------~~ |
| *Gorilla gorilla gorilla* | Western gorilla | Primates – Hominidae | XM_019024958.2 | Fc receptor like 2 (FCRL2) | ~~---------------~~ |
| *Pongo abelii* | Sumatran orangutan | Primates – Hominidae | XM_024234743.1 | Fc receptor-like protein 2 | ~~---------------~~ |
| *Nomascus leucogenys* | Northern white-cheeked gibbon | Primates – Hylobatidae | XM_003258642.4 | Fc receptor like 2 (FCRL2) | ~~---------------~~ |
| *Piliocolobus tephrosceles* | Ugandan red Colobus | Primates – Cercopithecidae | XM_023214131.3 | Fc receptor like 2 (FCRL2) | ~~---------------~~ |
| *Colobus angolensis palliatus* | Angola colobus | Primates – Cercopithecidae | XM_011928474.1 | Fc receptor-like 2 (FCRL2) | ~~---------------~~ |
| *Rhinopithecus roxellana* | Golden snub-nosed monkey | Primates – Cercopithecidae | XM_010381260.2 | Fc receptor-like protein 2 | ~~---------------~~ |
| *Rhinopithecus bieti* | Black snub-nosed monkey | Primates – Cercopithecidae | XM_017860071.1 | Fc receptor-like protein 2 | ~~---------------~~ |
| *Mandrillus leucophaeus* | Drill | Primates – Cercopithecidae | XM_011969338.1 | Fc receptor-like 2 (FCRL2) | ~~---------------~~ |
| *Chlorocebus sabaeus* | Green monkey | Primates – Cercopithecidae | XM_007976649.1 | Fc receptor-like 2 (FCRL2) | ~~---------------~~ |
| *Papio anubis* | Anubis baboon | Primates – Cercopithecidae | XM_003892856.5 | Fc receptor like 2 (FCRL2) | ~~---------------~~ |
| *Cercocebus atys* | Sooty mangabey | Primates – Cercopithecidae | XM_012078806.1 | Fc receptor-like 2 (FCRL2) | ~~---------------~~ |
| *Theropithecus gelada* | Gelada baboon | Primates – Cercopithecidae | XM_025392713.1 | Fc receptor-like protein 2 | ~~---------------~~ |
| *Macaca mulatta* | Rhesus monkey | Primates – Cercopithecidae | XM_001116902.4 | Fc receptor like 2 (FCRL2) | ~~---------------~~ |
| *Macaca fascicularis* | Crab-eating macaque | Primates – Cercopithecidae | XM_005541354.2 | Fc receptor like 2 (FCRL2) | ~~---------------~~ |
| *Macaca nemestrina* | Pigtail macaque | Primates – Cercopithecidae | XM_011769948.2 | Fc receptor-like protein 2 | ~~---------------~~ |
| *Cebus capucinus imitator* | Panamanian white-faced capuchin | Primates – Cebidae | XM_017503725.1 | Fc receptor like 2 (FCRL2) | ~~---------------~~ |
| *Callithrix jacchus* | Common Marmoset | Primates – Cebidae | ENSCJAT00000012925.4 | Fc receptor like 2 (FCRL2) | ~~---------------~~ |
| *Saimiri boliviensis boliviensis* | Bolivian squirrel monkey | Primates – Cebidae | XM_010348615.1 | Fc receptor-like protein 2 | ~~---------------~~ |
| *Carlito syrichta* | Philippine tarsier | Primates – Tarsiidae | XM_008062950.1 | Fc receptor-like protein 2 | ~~---------------~~ |
| *Microcebus murinus* | Gray mouse lemur | Primates – Cheirogaleidae | XM_012748726.1 | Fc receptor like 2 (FCRL2) | ~~---------------~~ |
| *Aotus nancymaae* | Ma's night monkey | Primates – Aotidae | XM_012449767.2 | Fc receptor like 2 (FCRL2) | ~~---------------~~ |
| *Propithecus coquereli* | Coquerel's sifaka | Primates – Indriidae | XM_012658484.1 | Fc receptor-like protein 2 | ~~---------------~~ |
| *Tupaia chinensis* | Chinese tree shrew | Scandentia – Tupaiidae | XM_014591823.2 | Fc receptor-like protein 2 | ~~---------------~~ |
| *Loxodonta africana* | Elephant | Proboscidea – Elephantidae | XM_023554129.1 | Fc receptor-like protein 2 | ~~---------------~~ |
| *Manis javanica* | Malayan pangolin | Pholidota – Manidae | XM_017650150.1 | Fc receptor-like protein 2 | ~~---------------~~ |
| *Oryctolagus cuniculus* | European rabbit | Lagomorpha – Leporidae | ENSOCUT00000009046.4 | Fc receptor like 2 | ~~---------------~~ |
| FCRL3 |  |  |  |  |  |
| *Homo sapiens* | Human | Primates – Hominidae | NM_052939.4 | Fc receptor like 3 (FCRL3) | ~~---------------~~ |
| *Bos Taurus* | European cattle | Artiodactyla – Bovidae | ENSBTAT00000001279.5 | Fc receptor like 3 | ~~---------------~~ |
| *Monodon monoceros* | Narwhal | Cetacea – Monodontidae | XM_029227202.1 | Fc receptor like 3 (FCRL3) | ~~---------------~~ |
| *Felis catus* | Cat | Carnivora – Felidae | ENSFCAT00000010049.6 | Fc receptor like 3 | ~~---------------~~ |
| *Oryctolagus cuniculus* | European rabbit | Lagomorpha – Leporidae | ENSOCUG00000009034 | Fc receptor like 3 | ~~---------------~~ |

**Supplementary Table 2.** Genome assembly and gene location for the sequences used to construct the synteny maps.

| **Species** | **Common name** | **Genome assembly** | **Chromosome** | **Location** |
| --- | --- | --- | --- | --- |
| *Mus musculus* | House mouse | GRCm39 (GCF_000001635.27) | 3 | NC_000069.7 (87158043..87171060, complement) |
| *Mus pahari* | Shrew mouse | PAHARI_EIJ_v1.1 (GCF_900095145.1) | 4 | NC_034593.1 (77780874..77791071, complement) |
| *Rattus norvegicus* | Norway rat | Rnor_6.0 (GCF_000001895.5) | 2 | NC_005101.4 (186594442..186605115, complement) |
| *Cavia porcellus* | Domestic guinea pig | Cavpor3.0 (GCF_000151735.1) | Unplaced Scaffold | NT_176363.1 (6022783..6034369) |
| *Loxodonta africana* | African savanna elephant | Loxafr3.0 | Unplaced Scaffold | SuperContig scaffold_33: 6,401,504-6,408,690 |
| *Sorex araneus* | European shrew | SorAra2.0 (GCF_000181275.1) | Unplaced Scaffold | NW_004546004.1 (2934161..3085392, complement) |
| *Otolemur garnettii* | Small-eared galago | OtoGar3 | Unplaced Scaffold | Scaffold GL873610.1: 2,715,035-2,723,070 |
| *Canis lupus familiaris* | Dog | CanFam3.1 (GCF_000002285.3) | 7 | NC_006589.3 (40479954..40491160, complement) |
| *Canis lupus dingo* | Dingo | UNSW_AlpineDingo_1.0 (GCF_012295265.1) | 7 Unlocalized Scaffold | NW_023365116.1 (41055851..41067054, complement) |
| *Vulpes vulpes* | Red fox | VulVul2.2 (GCF_003160815.1) | Unplaced Scaffold | NW_020356480.1 (13345732..13356825, complement) |
| *Ursus maritimus* | Polar bear | UrsMar_1.0 (GCF_000687225.1) | Unplaced Scaffold | NW_007907182.1 (2560951..2571745, complement) |
| *Ursus arctos horribilis* | Grizzly bear | ASM358476v1 (GCF_003584765.1) | Unplaced Scaffold | NW_020656218.1 (2491972..2502867, complement) |
| *Bubalus bubalis* | Water buffalo | ASM312139v1 (GCF_003121395.1) | 6 | NC_037550.1 (12075262..12121160, complement) |
| *Bos taurus* | Cattle | ARS-UCD1.2 (GCF_002263795.1) | 3 | NC_037330.1 (12437151..12451144, complement) |
| *Capra hircus* | Goat | ASM170441v1 (GCF_001704415.1) | 3 | NC_030810.1 (107605890..107624580) |
| *Eptesicus fuscus* | Big brown bat | EptFus1.0 (GCF_000308155.1) | Unplaced Scaffold | NW_007370735.1 (5468871..5485524) |
| *Phyllostomus discolor* | Pale spear-nosed bat | mPhyDis1.pri.v3 (GCF_004126475.2) | 14 | NC_040916.2 (449603..463529) |
| *Myotis lucifugus* | Little brown bat | Myoluc2.0 (GCF_000147115.1) | Unplaced Scaffold | NW_005871238.1 (422277..434250) |
| *Myotis davidii* | David’s bat | ASM32734v1 (GCF_000327345.1) | Unplaced Scaffold | NW_006291020.1 (210435..227562, complement) |
